# From Infancy to Modern Day: The History of Mother and Baby Units in the United Kingdom

**DOI:** 10.1192/bjo.2022.183

**Published:** 2022-06-20

**Authors:** Rui-Ernn Natassia Chin, Mao Fong Lim

**Affiliations:** 1 Camden and Islington NHS Foundation Trust, London, United Kingdom; 2 Cambridgeshire and Peterborough NHS Foundation Trust, Cambridge, United Kingdom

## Abstract

**Aims:**

Mother and baby units (MBUs) are inpatient units where women with severe acute postpartum psychiatric problems can be cared for alongside their babies. This is currently considered to be gold-standard care, recognising the importance of early childhood bonding and family-centered care. Great Britain has spearheaded the development of the MBU, however the history of MBUs in the United Kingdom (UK) has never been published.

**Methods:**

Through a narrative review of published and grey literature, we explore the development of the MBU in the UK, from its infancy to modern day.

**Results:**

We outline the history of the MBU model of care, from its early conception to current state. We also examine factors contributing towards the expansion of MBUs and more broadly, the expansion of perinatal mental health services throughout the UK. We also briefly describe the approach to MBUs worldwide, taking into consideration sociocultural differences and approaches to caring for the mother-baby dyad.

**Conclusion:**

Since its conception, there has been considerable investment in and expansion of perinatal mental health services, both in community and inpatient settings. Sustained research and continued advocacy is required to expand provision of care.

